# Analysis of NT-proBNP and uric acid due to left ventricle hypertrophy in the patients of aortic valve disease

**DOI:** 10.12669/pjms.35.1.148

**Published:** 2019

**Authors:** Muhammad Ishtiaq Jan, Riaz Anwar Khan, Aneesa Sultan, Anwar Ullah, Ayesha Ishtiaq, Iram Murtaza

**Affiliations:** 1 Muhammad Ishtiaq Jan, PhD. Signal Transduction Lab, Department of Bio-Chemistry, Faculty of Biological Sciences, Quaid-I-Azam University Islamabad, Islamabad, Pakistan; 2 Riaz Anwar Khan, MBBS, FCPS, MCPS. Department of Cardiovascular Surgery, Lady Reading Hospital Peshawar, Pakistan; 3 Aneesa Sultan, PhD Cancer Genetics Lab, Department of Bio-Chemistry, Faculty of Biological Sciences, Quaid-I-Azam University Islamabad, Islamabad, Pakistan; 4 Anwar Ullah, PhD. Cancer Genetics Lab, Department of Bio-Chemistry, Faculty of Biological Sciences, Quaid-I-Azam University Islamabad, Islamabad, Pakistan; 5 Ayesha Ishtiaq, MS. Signal Transduction Lab, Department of Bio-Chemistry, Faculty of Biological Sciences, Quaid-I-Azam University Islamabad, Islamabad, Pakistan; 6 Iram Murtaza, PhD. Signal Transduction Lab, Department of Bio-Chemistry, Faculty of Biological Sciences, Quaid-I-Azam University Islamabad, Islamabad, Pakistan

**Keywords:** NT-proBNP, Uric acid, Aortic valve, Heart failure, Left ventricle hypertrophy

## Abstract

**Objective:**

To evaluate the concentration of N terminal proBNP (NT-proBNP) and partially the serum uric acid in the severe condition of aortic valve dysfunction for assessment of left ventricle hypertrophy.

**Methods:**

The study was conducted in the signal transduction lab department of biochemistry Quaid-I-Azam University, Islamabad from September 2013 to February 2017. NT-proBNP and serum uric acid were measured in one hundred patients of aortic valve dysfunction. The patients were divided into three main groups: 1) Aortic stenosis, 2) Aortic regurgitation, and 3) Aortic stenosis with Aortic regurgitation. The results were compared between disease and controls groups.

**Results:**

High level of plasma NT-proBNP was detected in all the three disease groups of aortic valve (stenosis, p<0.001), (regurgitation, p<0.001) and (stenosis with regurgitation, p<0.001). In addition, non-significantly increased level of serum uric acid was also observed in left ventricle hypertrophy in all the three respective disease groups of aortic valve.

**Conclusion:**

Increased secretion of NT-proBNP during cardiac remodeling can be related to the severity of left ventricle hypertrophy due to aortic valve abnormality in all the disease groups of severe stenosis, severe regurgitation, and combine disease condition of severe stenosis and severe regurgitation. However, non-significant increase in uric acid concentration is also identified which may be due to one of the factors involved in left ventricle hypertrophy in all the three disease groups of aortic valve. The interaction of uric acid with NT-proBNP during cardiac remolding due to aortic valve dysfunction is still not clear.

## INTRODUCTION

Brain natriuretic peptide (BNP) is a member of the natriuretic peptide family.^1^ The synthesis and secretion of BNP mostly occur in ventricular myocardium. Initially, the synthesis of BNP is occurred as a prehormone (proBNP) which comprise of 108 amino acids. After its release in the circulation it is then cleaved into biologically active C-terminal fragment 32 amino acid BNP and biologically inactive N-terminal fragment 76 amino acid (NTproBNP). Both molecules can be used as potent cardiac hypertrophic markers in blood.^2^ The secretion of BNP is increased in heart failure (HF). Patients have elevated level of plasma BNP effected from systolic left ventricular dysfunction (LVD) including hypertrophy and HF. Moreover, in clinical practice BNP based diagnosis provides supportive information for the identification of LVD on the basis of history, echocardiography, Chest X Ray and physical examination.^3^ Therefore BNP levels can be used as a good platform for the evaluation of LVD due to hypertrophy. In diastolic dysfunction and HF, BNP can be used as a potent marker. Moreover, in cardiac hypertrophy NT-proBNP is considered a discerning marker. Recently, BNP get more attention as a suboptimal marker for detection of diastolic dysfunction. Furthermore, it has also been investigated that BNP is also high in patients with aortic and mitral valve disease due to pressure overload in ventricles. Numerous population based studies revealed that elevated level of uric acid may be a risk of cardiovascular mortality.^4^ The increase level of uric acid is mostly found in metabolic disorder. However, the high level of uric acid is also associated with the age, gender, and other factors. According to recent reports increased production of serum uric acid through activation of xanthine oxidase (XO) from xanthine and hypoxanthine was found in the patients of chronic heart failure (CHF).^5^ XO also generates free radicals including reactive oxygen species (ROS) which may participate in oxidative damage in the myocardium.^6^ However, ROS also play a crucial role during cardiomyocytes apoptosis.^7^ In addition, inadequate ROS levels were also found in human cardiac valve dysfunction during apoptosis. The detail study with animal models showed that high level of uric acid ultimately leads to mitochondrial DNA damage due to oxidative stress.^8^ In chronic condition of myocardial oxidative stress, it can further causes subcellular abnormalities, and may finally leads to HF. It reveals that increase level of serum uric acid may contribute to cardiac dysfunction by increased XO activity. The increase in concentration of NT-proBNP^2^ and uric acid^4^ shows a positive co-relationship with left ventricular hypertrophy due to aortic valve dysfunction. However, regression analysis shows a weak corelationship between the two markers. It is also unknown that uric acid independently can provide useful information during HF which needs further research.

In current study, we hypothesized that NT-proBNP and uric acid are increased due to pressure overload in left ventricle hypertrophy due to aortic valve dysfunction. The increase levels of uric acid are might be one of the factors due to left ventricle hypertrophy. Therefore, we have determined the plasma NT-proBNP and serum uric acid in aortic valve patients, to characterize the extent of left ventricle hypertrophy.

## METHODS

The study was conducted in the signal transduction lab department of biochemistry Quaid-I-Azam University, Islamabad from September 2013 to February 2017. The patients were recruited from Department of Cardiovascular Surgery, Lady Reading Hospital (LRH) Peshawar. The institutional ethics and clinical research committee of LRH Peshawar and Quaid-I-Azam University, Islamabad, have approved this study. All the patients were given informed consent before participating in study. The patients were categorized into three main groups on the basis of echocardiographic results: 1) Aortic stenosis, 2) Aortic regurgitation, and 3) Aortic stenosis with Aortic regurgitation.

The patients of aortic valve disease with associated mitral valve disease, coronary heart disease, renal dysfunction, cerebrovascular accident, and associated congenital heart diseases were excluded from the present findings.

Age and gender matched controls were recruited at the time of patients recruitment. In addition, regression analysis were performed to neutralize the effect of age between disease and control groups (Supplementary Fig.1). In the current investigation hundred patients were selected of only aortic valve dysfunction including ninety six males and four females with mean age 43.6±14.1 years (50.7±17.3 and 36.6±11 years respectively). Data was also collected from hundred healthy individuals (fifty males and fifty females with mean age 44.6±13.2 years (46.7±13.5 and 42.5±12.9 years respectively). The severity of chronic heart failure was accessed as by New York Heart Association (NYHA) class III in ninety four and class IV in six patients. All the patients were in severe stage of valve dysfunction. These patients were admitted for the surgery of valve replacement in cardiovascular department of LRH Peshawar. The initial diagnosis and disease status of already operated patients were studied from patient’s diagnostic history which was helpful for proposed reason and severity of disease for surgery. In addition, blood samples were collected from all the patients in fasting state.

**Fig.1:**
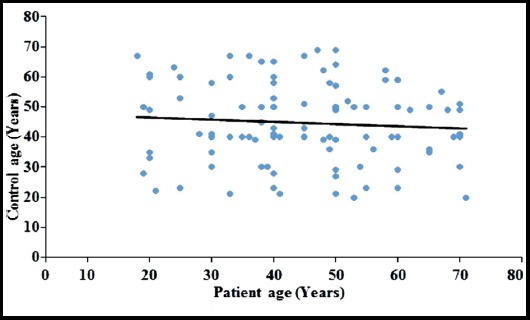
The scattered diagram shows that patients and healthy age. The trend line of regression is horizontal showing the neutralization of age effect between control and disease groups.

### Base line diagnosis and severity of valve disease

Chest X ray and CT scan were performed for initial diagnosis and evaluation of thoracic and extra thoracic malignancy or tumor and pulmonary embolism and function. During image interpretation, a standard five point scale was used for different grades of calcification.^9^ The severity of cardiac valves was observed through standard criteria of thickening, regurgitation, ejection fraction, and area of aortic valves during transthoracic echocardiography (TTE). In addition, a standard three point scale was used for regurgitation.^10^ Angiography was also performed for the patients with age greater than 35 years.

### Biochemical Analysis

Blood was collected from the patients in supine resting position for a minimum time period of 30 minutes. The blood samples were chilled and centrifuged for NT-proBNP measurements. The measurement of NT-proBNP was performed with fluorescence immunoassay using the instruction provided with kit (Aviva system biology, California, Cat # OKCA00041). Serum uric acid was measured with commercially available kit (Cat # MAK077-1KT, Merck) using chemistry analyzer (Model microlab 200, Merck).

### Statistical Analysis

Data were analyzed by SPSS 21 (IBM, USA). Statistical tests ANOVA followed by multiple comparison tests such as Tukey’s post-hoc test were performed to evaluate the significant difference among the groups. All the data were presented as mean ± SD or SEM. *P* value was statistically significant when less than 0.05.

The sample size was calculated as the following formula and the total sample size was found to be 198.86.

Sample size = r+1/r × SD² (Zß + Zα/2)²/d^2^

R = ratio of control to cases, 1 for equal number of case and control

SD = Standard deviation of ±4.5 was considered.

D = Expected mean difference between case and control

Zß = Standard normal variate for power = for 80% power of study it is 0.84

(Zα/2): standard normal variate for level of significance

SPSS 21 (IBM Corporation, USA) was used for Statistical analysis. *P* value was consider significant when less than 0.05. All the basic characteristics and parameters were presented as mean ± SD.

## RESULTS

### NT-proBNP in patients of aortic valve disease

In chronic condition of left ventricle hypertrophy due to aortic valve dysfunction the plasma NT-proBNP level is increased due to pressure overload in ventricle.^11^ In individuals of the control group NT-proBNP plasma concentration was 24.7±8.5 pg/ml. In patients with aortic stenosis (AS) the concentration of NT-proBNP was significantly high (5990.2±5893 pg/ ml) (p< 0.001 vs. control). Those patients with aortic regurgitation (AR) have also presented a significantly high value of NT-proBNP (5375.8±4220 pg/ml) (p<0.001 vs. control). In addition, the patients with both the characteristics of aortic stenosis and regurgitation also showed a significantly high level of NT-proBNP (2201.2±804 pg/ml) (p<0.001 vs. control) given in Table-I and Table-II.

**Table-I:** Comparison of clinical (levels of NT-proBNP and uric acid) and demographic parameters in sub types of disease and controls.

	Sub Types of Disease		

	AS	AR	AS+AR
Patients	6	8	86
Age (Years)	55.9±11.4	29.8±17.2	44.7±13.7
Gender (M/F)	4/2	8/0	84/2
NT-proBNP (pg/ml)	5990.2±5893	5375.8±4220	2201.2±804
*P* value	<0.001*	<0.001*	<0.001*
Serum Uric Acid (mg/dl)	6.4±1.9	5.6±1.3	6.1±1.2
*P* value	0.94	0.86	0.14
Controls	100		
Age (Years)	Male (46.7±13.5) Female (42.5±12.9)		
Gender (M/F)	1/1		
NT-proBNP (pg/ml)	24.7±8.5		
Serum Uric Acid (mg/dl)	4±0.1.4		

δAortic valve (A), Stenosis (S), Regurgitation (R), N-terminal pro-brain natriuretic peptide (NT-proBNP)

* Tukey multiple comparison test, *P value was significant when less than 0.05

**Table-II:** Clinical (levels of NT-proBNP and uric acid) and demographic parameters in aortic valve disease groups and controls.

Sub groups of aortic valve disease		NT-proBNP			Uric Acid		

		(pg/ml) ± SD	Mean Difference at 95% Confidence Interval	*P* Value	(mg/dl) ± SD	Mean Difference at 95% Confidence Interval	*P* Value
AS+AR (n = 86)	AS	5990.2±5893	-3788.9	<0.001*	6.4±1.9	2.1	0.85
	AR	5375.8±4220	-3174.6	<0.003*	5.6±1.3	1.6	0.89
	Control	24.7±8.5	2176.4	<0.001*	4±0.1.4	3.6	0.14
AS (n = 6)	AS+AR	2201.2±804	3788.9	<0.001*	6.1±1.2	-2.1	0.85
	AR	5375.8±4220	614.2	0.955	5.6±1.3	-0.45	0.9
	Control	24.7±8.5	5965.3	<0.001*	4±0.1.4	1.5	0.94
AR (n = 8)	AS+AR	2201.2±804	3174.6	<0.003*	6.1±1.2	-1.6	0.89
	AS	5990.2±5893	-614.2	0.955	6.4±1.9	0.45	0.9
	Control	24.7±8.5	5351.1	<0.001*	4±0.1.4	2.0	0.86
Control (n=100)	AS+AR	2201.2±804	-2176.4	<0.001*	6.1±1.2	-3.6	0.14
	AS	5990.2±5893	-5965.3	<0.001*	6.4±1.9	-1.5	0.94
	AR	5375.8±4220	-5351.1	<0.001*	5.6±1.3	-2.0	0.86

δAortic valve (A), Stenosis (S), Regurgitation (R), N-terminal pro-brain natriuretic peptide (NT-proBNP)

* Tukey multiple comparison test, *P* value was significant when less than 0.05.

### Uric acid level in patients of aortic valve disease

**Fig.2:**
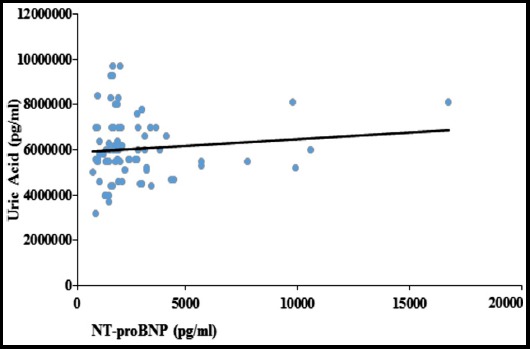
The diagram shows the relationship between NT-proBNP and Uric Acid. The trend line of regression shows a weak co-relationship between the two markers.

Numerous previous investigations revealed that elevated level of uric acid is involved in various cardiovascular diseases including ventricle hypertrophy.^4^ However, the exact mechanism of high uric acid with increased incidence of various cardiac complications is still unclear. In our study, the level of uric acid in control individuals was 4±0.1.4 mg/dl. However, level of uric acid was 6.4±1.9 mg/dl (p<0.94 vs. control) in the patients of AS. In the patients of AR, the level of uric acid was 5.6±1.3 mg/dl (p<0.86 vs. control). Those patients who have both the disease condition of aortic stenosis with regurgitation have 6.1±1.2 mg/dl (p<0.14 vs. control) concentration of uric acid. All the three groups of aortic valve disease showed a non-significant increase of uric acid concentration given in Table-I and Table-II. In addition, regression analysis showed a weak corelationship between NT-proBNP and uric acid. Fig.2.

## DISCUSSION

Cardiac valve dysfunction is associated with human heart complications. This may also leads to chronic heart failure due to volume or pressure overload of ventricles which further causes left ventricle hypertrophy. The severity of ventricle hypertrophy due to cardiac valve dysfunction can be evaluated by the level of BNP^13^,^14^ and NT-proBNP.^11^ However, among different cardiac markers NT-proBNP can provides better informations as compared to BNP. In the present investigations we have found a significant high level of NT-proBNP expression in all the three pathological conditions of stenosis and regurgitation of aortic valve with left ventricle hypertrophy justifying the previous data.^15^ In addition, the significant high level of NT-proBNP also showed increase in severity of left ventricular hypertrophy in all the disease groups. In aortic valve patients the left ventricle increases the secretion of NT-proBNP level due to increase in pressure overload.^11^ Several other studies have illustrated that NT-proBNP is associated with functional status and progression of cardiac hypertrophy.^2^ The diagnostic utility data of NT-proBNP regarding left ventricle hypertrophy due to aortic regurgitation is very limited but it is demonstrated that NT-proBNP is increased during disease severity. The evidences have showed that in future the assessment of NT-proBNP can be helpful in diagnosis and severity of cardiac hypertrophy due to aortic valve disease which needs further investigations. In addition to NT-proBNP, serum uric acid level was also increased during stenosis, regurgitation and both the disease conditions of stenosis and regurgitation of aortic valve due to left ventricular hypertrophy. However, regression analysis revealed a weak co-relation between both the markers. The level of uric acid also increases during cardiovascular disease which might be due to the increased production of XO enzyme from myocardium during hypertrophy. The conversion of hypoxanthine to uric acid is regulated by XO.^16^ However XO is produced by liver and small intestine but evidence showed that XO can also be produced locally by myocardium in many cardiac disease related to the oxidative stress such as left ventricular remodeling.^17^ ROS can also be generated by XO which cause cytotoxicity in physiological and pathological conditions.^18^ However, ROS can also damage the myocardial tissue due to the increased rate of apoptosis and other cardiac complications.^19^-^21^ In-vivo, the increase level of uric acid in rats damage the mitochondrial DNA and causes oxidative stress.^8^ Hyperuricaemia is commonly observed in cardiovascular disease however it cannot be considered as a true risk factor because it may also vary according to age and gender.^22^ On the contrary, some other factors including obesity, diet, and metabolic syndrome can also lead to elevated level of uric acid. In present study, to overcome the effect of diet on the concentration of uric acid the blood samples were collected in fasting state.^23^,^24^ Herein, we demonstrated that non-significant increase in uric acid may also be due to the left ventricle hypertrophy caused by the aortic valve disease. Advance studies have showed that oxidative stress, vasoconstriction, and inflammation can also be stimulated by uric acid.^25^ However, further studies are required on hyperuricaemia and its association with pathophysiology in cardiovascular diseases.

### Limitations of the study

In present study the samples were collected only from Lady Reading Hospital (LRH) Peshawar. The data was analyzed in one ethnic group of patients (Pushton). The sample size was also relatively small.

## CONCLUSION

The significantly high level of NT-proBNP was observed during the severe conditions of aortic valve disease due to stenosis, regurgitation and both pathological conditions. Furthermore, non-significant increased level of uric acid was also observed in all the disease conditions of aortic valve. Moreover, partially uric acid level may also be contributed by left ventricle hypertrophy. The high level of NT-proBNP concentrations can provide useful information about the severity of left ventricle hypertrophy due to aortic valve abnormalities However, independent increase of uric acid and their relationship with NT-proBNP during left ventricle hypertrophy is not clear which needs further investigation.
